# Developmental differences in platelet P-selectin expression modulate neonatal monocyte inflammatory responses

**DOI:** 10.1080/09537104.2026.2726287

**Published:** 2026-09-22

**Authors:** Erin Soule-Albridge, Emily Flahardy, Henry A. Feldman, Natalie Kane, Kimberley Banfield, Amalia El Nems, Jesselin Romero Escobar, Martha Sola-Visner, Patricia Davenport

**Affiliations:** 1 Boston Children’s Hospital, Division of Newborn Medicine, Boston, MA, USA; 2 Department of Pediatrics, Harvard Medical School, Boston, MA, USA; 3 Biostatistics and Research Design Center, Boston Children’s Hospital, Boston, MA, USA

**Keywords:** Monocyte, neonate, transfusion

## Abstract

Neonatal and adult platelets are functionally different, with neonatal platelets being comparatively hypo-reactive to multiple platelet agonists. When transfused, neonates receive platelets from adult donors, resulting in a cellular developmental mismatch. Platelets are key regulators of the monocyte inflammatory response, yet how the developmental stage of the platelet affects this response is unknown. In this study, we modeled this developmental mismatch *in vitro* to investigate the neonatal (cord blood) monocyte inflammatory response and migratory phenotype after exposure to either neonatal (cord blood) or adult platelets. Adult platelets expressed more P-selectin on the surface and released more beta-2-microglobulin (B2M) after activation compared to neonatal platelets. While exposure to platelets of either developmental stage increased the release of IL-8 and MCP-1 from neonatal monocytes to a similar degree, exposure to adult platelets induced significantly greater monocyte CCR2 and CCR5 surface expression, consistent with a pro-migratory monocyte phenotype. Blocking P-selectin/PSGL-1 interactions had no effect on platelet induced monocyte cytokine release but completely abrogated monocyte CCR2 and CCR5 surface upregulation. These findings suggest that the monocyte pro-migratory phenotype is regulated by P-selectin exposure, which is influenced by the platelet developmental stage, while monocyte cytokine release is independent of platelet P-selectin expression or developmental stage.

## PLAIN LANGUAGE SUMMARY

**What is the context?:**

- Premature neonates in the neonatal intensive care unit (NICU) often receive platelet transfusions to prevent bleeding, but use of higher transfusion thresholds has been linked to increased death, bleeding, lung disease, and developmental delays.
- This may be due to a “mismatch” between the neonate’s platelets and the transfused platelets from adult donors. Compared to neonatal platelets, adult platelets are more active and potentially more inflammatory.
- Platelets help regulate inflammation through interactions with immune cells like monocytes, but how exposure to neonatal vs. adult platelets affects the monocyte inflammatory response is unknown.

**What is new?:**

- In this study, we found that monocytes isolated from neonates responded differently after exposure to adult vs. neonatal platelets.
- Exposure to adult platelets increased CCR2 and CCR5 on monocytes, priming them to migrate from blood into tissue – an effect driven by platelet P-selectin binding to the monocyte receptor PSGL-1.

**What is the Impact?:**

- These findings show the need to better understand how platelet immune function changes with development, so transfusion products can be better matched to a patient’s age – reducing harmful inflammation and improving outcomes.

## Introduction

Infants admitted to the neonatal intensive care unit (NICU), and especially those born preterm, have a high rate of both thrombocytopenia (platelet count <150,000/μL) and severe bleeding.^1,2^ As a result, they frequently receive platelet transfusions at higher platelet count thresholds than older children or adults in hopes of preventing significant hemorrhage.^3–6^ The benefit of these transfusion practices was recently questioned by the results of the PlaNet-2 study (Platelets for Neonatal Transfusion Trial), which found that premature neonates randomized to receive a platelet transfusion at a higher platelet count threshold had a higher incidence of mortality and/or major bleeding as well as bronchopulmonary dysplasia (chronic lung disease of prematurity) compared to neonates randomized to transfusion at a lower platelet count threshold.^7^ Furthermore, infants randomized to the high threshold were also found to have a higher incidence of death and/or poor neurodevelopmental outcomes and higher need for respiratory support at 2 years corrected age.^8^ While increased morbidity and mortality associated with platelet transfusions has been described in selected adult patient populations,^9–11^ preterm neonates appear to be uniquely vulnerable to platelet transfusion-related harm. This vulnerability may be due, at least in part, to the developmental mismatch that occurs when neonates receive a transfusion of donated adult platelets.

Neonatal, compared to adult, platelets are hypo-reactive to many platelet agonists and express comparatively less P-selectin on their surface after activation.^12^ While most developmental differences in platelet function have been investigated in regard to platelet hemostatic functions, platelets are now recognized as key participants in and active regulators of immune and inflammatory responses.^13,14^ Platelets interact with immune cells through direct cell–cell interactions as well as through the release of soluble mediators. Upon activation, P-selectin expressed on the platelet surface is available to interact with its receptor (PSGL-1) located on multiple cell types including neutrophils and monocytes. Platelets also store and release immunomodulatory proteins including sCD40L, platelet factor 4 (PF4), RANTES (CCL5), NAP-2 (CXCL7), and beta 2 microglobulin (β2 M).^15–20^ Through a combination of direct and indirect interactions, platelets have been found to regulate monocyte inflammatory responses^17,18,21–23^ and promote neutrophil activation, migration and extracellular trap (NET) release.^24–35^

Until recently, all studies investigating the immune and inflammatory functions of platelets had been conducted using adult cells and adult models of disease. Thus, little was known about how the immune functions of platelets change over development and whether/how differences in immune/inflammatory functions between neonatal and adult platelets potentially contribute to the harm associated with neonatal platelet transfusions.

In a recent study comparing the platelet proteome between neonates and adults we found adult platelets to be enriched in proteins involved in immune regulation and inflammation, while neonatal platelets were enriched in proteins related to ribosomal components and metabolic activities.^36^ These findings suggested that adult platelets may be more immunologically active than neonatal platelets. Consistently, Maurya et al. found that transfusion of either neonatal or adult platelets into thrombocytopenic newborn mice increased pro-inflammatory (Ly6C^hi^) monocytes, but only the transfusion of adult platelets increased the percentages of circulating monocytes expressing CCR2 and CCR5, the receptors for two potent chemokines (MCP-1 and RANTES, respectively).^37^ The presence of these receptors conferred a pro-migratory phenotype on neonatal monocytes *exposed to adult platelets*, enabling them to more efficiently migrate in response to MCP-1 and RANTES gradients. However, it is unknown whether neonatal and adult platelets have similar differential effects on human neonatal monocytes. In the current study, we exposed primary human neonatal (cord blood) monocytes to either activated adult or neonatal platelets to investigate whether and how the “developmental mismatch” that occurs when neonates are transfused with adult platelets alters the immune responses of human neonatal monocytes.

## Methods

### Human subjects

This study was approved by the IRB of both Boston Children’s Hospital and Beth Isreal Deaconess Medical Center (BIDMC), IRB #P00023047 and #X09–11-0605. Cord blood (CB) was collected from the placentas of healthy full-term infants born via scheduled cesarean section at BIDMC and brought to the lab immediately after collection. Simultaneously, adult blood (AB) was collected by venous blood draw from healthy volunteers who had not taken any anti-platelet medication in the preceding 7 days. Both blood samples were collected into 30 mL syringes containing 5 mL of acid citrate dextrose (ACD) with a 21 G needle. Both blood samples were processed in tandem, within 30 minutes after collection.

### Platelet isolation

Platelets from both CB and AB samples were isolated using the platelet rich plasma (PRP) method. All platelet centrifugation steps were done without acceleration or brake. Whole blood was first centrifuged at 250 g for 15 min. PRP was removed and, after the addition of 100 nM PGE-1 (Sigma Aldrich; #P5515), the sample was centrifuged at 120 g for 10 min to remove any remaining red or white blood cells. The pure PRP was then removed and after the addition of 100 nM PGE-1, was centrifuged at 400 g for 30 min to pellet the platelets. Pelleted platelets were washed in PIPES/saline/glucose (5 mM PIPES, 145 mM NaCl, 4 mM KCl, 50 μM Na_2_ HPO_4_, 1 mM MgCl_2_-6 H_2_O, and 5.5 mM glucose) with 100 nM PGE-1. The final platelet pellet was resuspended in phenol red free Medium 199 (M199) (Gibco; #11043023) to a final concentration of 250,000 platelets/μL, confirmed by measuring the platelet count using a Sysmex Hematology Analyzer (XNV-1000).

### Monocyte isolation

CB Monocytes were isolated by Ficoll gradient and positive (CD14+) selection. After removal of PRP for the platelet isolation, the red and white blood cell component was diluted 1:1 with PBS and layered over Ficoll (Cytiva; #17–1440-02) in a blood to Ficoll ratio of 2:1. Next, the sample was centrifuged at 450 g for 30 min without acceleration or brake. The middle mononuclear cell layer was removed and centrifuged separately at 1250 g for 30 min to pellet the cells. The cellular pellet was washed once and pelleted again at 400 g for 10 min. The final pellet was then resuspended in 200–500 μL of PBS with 20–50 μL of CD14 microbeads (Miltenyi; #130–050-201) and incubated for 15 min at 4°C. Cells were then washed again with HBSS buffer (HBSS, 1% BSA, and 2 mM EDTA) to remove excess beads. The cells were then resuspended in the same HBSS buffer and passed through a magnetic MS column (Miltenyi; #130–042-2011) twice to isolate pure CD14+ cells (monocytes). Purified monocytes were pelleted and resuspended in M199 to a final concentration of 10 000 monocytes/μL confirmed by measuring the monocyte count using a Sysmex Hematology Analyzer (XNV-1000).

### Platelet activation status and cell purity

Platelet P-selectin surface expression was determined by flow cytometry immediately after washing (resting platelets) and after exposure to thrombin (activated platelets). Resting and activated platelets were incubated with CD41-APC (BD; #559777) and CD62P-PE (BioRad; #MCA2419PE). An isotype control (BioRad; #MCA928) served as the negative control (Supplemental Figure 1A). P-selectin median fluorescent intensity (MFI) was recorded and compared. Platelet purity was determined by incubating washed platelets with CD41-APC and CD45-FITC (BD; #555483) to confirm the absence of leukocyte contamination (Gating strategy is shown in Supplemental Figure 1B). Monocyte purity was determined by incubating purified monocytes with CD14-APC (BD; #340436) and CD45-FITC to confirm the absence of other leukocytes (Supplemental Figure 1C). Cells were stained for 15 min and fixed with 1% formalin. Samples were run on the LSRFortessa II. All data was analyzed using FlowJo.

### Platelet monocyte co-culture

400 μL of CB or AB platelets were placed in wells and either left resting or activated with 0.05 U/mL thrombin (Sigma-Aldrich; #605195) for 20 min. Platelet activation was stopped by adding 0.05 U/mL hirudin (Sigma-Aldrich; #H0393) for 20 min. As a control, monocytes were added to empty wells and exposed to identical doses of thrombin and hirudin. For experimental conditions, 100 μL of Monocytes were added to wells containing either activated or resting CB or AB platelets. The final cellular concentration in the wells was 200,000 platelets/μL and 2,000 monocytes/μL, as described previously.^21^ Cells were then co-cultured at 37°C until the pre-determined end points. After 4 h of co-culture, the cellular suspensions from one set of wells were removed for quantification of monocyte surface receptor expression by flow cytometry. After 18 h, the remaining cell suspensions were removed and centrifuged at 2800 g for 10 min and the supernatant aliquoted and stored at −80°C for analysis of monocyte cytokine release by ELISA.

### Monocyte surface receptor expression

After 4 h of incubation, each monocyte-platelet co-culture suspension was centrifuged at 200 g for 10 min to pellet the cells. Cells were then resuspended in PBS and incubated with 25 μg/mL FC block (BD; #564220) for 10 min. Cells were then incubated with CD192 (CCR2)-PE (BioLegend; #357206), CD195 (CCR5)-FITC (BD; #555992), and CD14-APC. Isotype controls for CCR2 (BioLegend; #400213) and CCR5 (BD; #555573) served as the negative controls. Samples were incubated for 15 min and fixed with 1% formalin. The data was analyzed using FlowJo. The gating strategy is shown in Supplemental Figure 1D.

### Cytokines measurement

Platelet and monocyte released cytokines concentrations were measured in supernatants collected from either platelets alone (resting and after activation) or cellular co-cultures by ELISA, performed according to manufacturer instructions. ELISA kits included β2 M (Invitrogen; #EHB2M), CCL5/RANTES DuoSet (R&D; #DY278), TGF-β1 DuoSet (R&D; #DY240), CCL2/MCP-1 DuoSet (R&D; #DY279), and IL-8/CXCL8 DuoSet (R&D; #DY208).

### PSGL-1 block

Platelets were activated as described. Monocytes were incubated with 50 μg/mL NA/LE FC block (BD; #564765) for 10 min then with 10 μg/mL anti-CD162 (PSGL-1) (BD;#556052) or an isotype control (BD; #554721) for 30-min prior to addition to platelets. Monocytes were then added to resting or activated platelets at the same concentrations as described previously. Outcomes were measured at the same 4 h and 18-h timepoints.

### Addition of recombinant β2 M

Monocytes were incubated with recombinant beta2microglobulin (β2 M) (Antibodies online; ABIN7533951) at different concentrations (40, 2000, 10 000 ng/mL). Monocytes were incubated with the recombinant β2 M for 4 and 18 h for analysis of identical timepoints described above.

### Statistical analysis

We employed factorial analysis of variance (ANOVA) to compare cytokine release and surface receptor expression in monocyte cultures exposed to platelets from cord blood (CB) or adult blood (AB). Besides the source of platelets, the ANOVA accounted for other conditions such as platelet activation, receptor blocking, and β2 M concentration, as well as day-to-day random variation among replicate experiments. From parameters of the fitted ANOVA model we constructed contrasts of interest among the ANOVA factors and limited statistical testing in the ANOVA to hypotheses formed *a priori*. Outcome variables were log-transformed for analysis to reduce skew and retransformed for reporting and displayed as adjusted mean and standard error (SE). To reduce the influence of extreme values we applied iterative reweighting, a statistical approach also termed “robust regression” used to identify outliers based on the magnitude of deviation of each datapoint from the fitted model.^38^ To assess some associations among the outcome measures, we used Pearson correlations (r) and log–log linear regression. SAS software (version 9.4, Cary, NC) was used for all statistical computations.

## Results

### Developmental differences in platelet P-selectin surface expression and soluble mediator release

In a first set of experiments, we compared P-selectin surface receptor expression between paired CB (*n* = 8) and AB (*n* = 8) platelets both immediately after washing (resting) and following thrombin activation (Figure 1(A)), and confirmed platelet and monocyte purity. Consistent with prior studies, we found that thrombin-activated CB platelets expressed less P-selectin on their surface compared to activated AB platelets (CB: 1987 ± 816 (MFI ± SE); AB: 4010 ± 1465; *p* = .04) with no developmental differences between resting platelets (CB: 371 ± 152; AB: 493 ± 202; *p* = .37) (Figure 1(B)). Next, levels of β2 M, TGF-β, and RANTES were measured in the releasates of the same resting and thrombin-activated platelets. β2 M concentrations were significantly higher in the releasates from AB compared to CB platelets, both at rest (AB: 18.0 ± 4.8 (ng/mL ± SE); CB: 12.1 ± 3.3; *p* = .03) and following thrombin activation (AB: 37.9 ± 10.2; CB: 17.9 ± 5.0; *p* = .0004) (Figure 1(C)). RANTES and TGF-β were also present at higher concentrations in the releasates of activated compared to resting platelets but there were no significant differences between neonatal and adult platelets in either condition (Figure 1(D, E)).

### Effects of platelets on IL-8 and MCP-1 release from CB monocytes

Monocytes isolated from the same CB samples described above plus an additional 4 new collections (total *n* = 12) were co-cultured with the well-characterized thrombin-activated CB and AB platelets shown in Figure 1 or with thrombin alone (control), and culture supernatants were collected after 18 h. Co-culture with platelets of either developmental origin significantly stimulated the release of IL-8 and MCP-1 from CB monocytes, compared to thrombin exposed monocytes (Figure 2(A)), with no significant differences between CB and AB platelets. Given the observed trend toward higher IL-8 and MCP-1 production in cultures with AB platelets, however, we then explored whether the P-selectin surface expression level or the concentration of β2 M, RANTES or TGF-β released by the platelets correlated with the level of IL-8 or MCP-1 production from neonatal monocytes. On a secondary exploratory regression analysis (Table I), we found a significant association between the platelet P-selectin surface expression and the level of IL-8 released by monocytes (slope of log IL-8 vs. log P-selectin = 0.69 ± 0.12 (SE), *p* = .001). The β2 M amount released by the platelets was also associated with the monocyte IL-8 production (slope of log IL-8 vs. log β2 *M* = 0.60 ± 0.22 (SE), *p* = .04) (Table I).

### Effects of platelets on the monocyte trafficking phenotype

In addition to releasing inflammatory cytokines, monocytes migrate to areas of inflammation in response to chemokine gradients. This pro-migratory phenotype is determined by the expression of chemokine receptors on the monocyte surface, such as CCR2 (the receptor for MCP-1) and CCR5 (the receptor for RANTES). Platelets were recently shown to stimulate CCR2 and CCR5 expression of the surface of neonatal *mouse* monocytes in a P-selectin-dependent manner.^37^ To determine whether the same effects were evident in a human system, we measured CCR2 and CCR5 expression on CB monocytes (isolated from 7 new CB samples) exposed to thrombin-activated CB or AB platelets (from 7 new adult donors). Platelets of both developmental origins increased monocyte CCR2 and CCR5 surface expression compared to controls, but AB platelets induced significantly higher expression levels of CCR5 (AB: 5614 ± 2537; CB: 3921 ± 1765; *p* = .001) (Figure 3(A,B)) and to a lesser extent CCR2 (AB: 23 504 ± 3085 (MFI ± SE); CB: 19 012 ± 2417; *p* = .02) (Figure 3(C,D)). Furthermore, the platelet P-selectin surface expression level strongly correlated with the monocyte surface expression of CCR2 (*r* = 0.53, *p* = .006) and particularly CCR5 (*r* = 0.79, *p* < .0001) after co-culture (Figure 3(E,F)). Importantly, we confirmed that CCR2 and CCR5 were not significantly expressed on the surface of activated platelets, ruling out the possibility that the detected signals came from platelets adhered to the cultured monocytes.

### Blocking P-selectin/PSGL1 interactions

To further investigate P-selectin’s role in monocyte cytokine release and surface receptor expression, we blocked the binding of platelet P-selectin to its receptor PSGL-1 on monocytes (isolated from 8 new CB samples) using a blocking anti-PSGL-1 antibody. As seen in Figure 4(A–D), blocking P-selectin did not significantly reduce the release from CB monocytes co-cultured with activated AB platelets (from 8 new adult donors) of either IL-8 (+anti-PSGL1: 1007 ± 377(% of control ± SE); +IgG: 1648 ± 599; *p* = .14) or MCP-1 (+anti-PSGL1: 615 ± 302; +IgG: 1275 ± 620; *p* = .09), but it significantly reduced the monocyte surface expression of CCR5 (+anti-PSGL1: 1268 ± 176; +IgG: 2119 ± 295; *p* < .0001) and -to a lesser degree- of CCR2 (+anti-PSGL1: 5217 ± 732 (MFI ± SE); +IgG: 6185 ± 868; *p* < .0001). The reasons why the addition of adult platelets did not increase CCR2 surface expression in these set of studies are unclear, but we hypothesized that the IgG control antibody might have blunted the CCR2 response.

### Addition of recombinant β2 M

Next, we investigated the role of β2 M in these same processes. In the absence of an effective human β2 M blocking antibody, we tested the effects of escalating concentrations of recombinant β2 M (rβ2 M) on CB monocytes. The concentrations tested were chosen to mimic the β2 M content measured in the releasate of adult activated platelets (40 ng/mL, Figure 1(B)) and concentrations previously shown to stimulate monocyte production of inflammatory cytokines (2,000 and 10 000 ng/mL).^18^ Addition of rβ2 M at the tested concentrations had no effect on monocyte IL-8 or MCP-1 release, nor did it increase CCR2 or CCR5 surface expression (Supplemental Figure 2A–D).

## Discussion

In this study, we found key differences in the immune responses of neonatal cord blood- derived monocytes after exposure to adult versus neonatal platelets. Compared to neonatal platelets, adult platelets are hyper-responsive to most platelet agonists and express more P-selectin on their surface following activation.^12^ Consistent with prior reports, we found developmental differences in thrombin-induced P-selectin surface expression, with higher levels on activated adult vs. neonatal platelets. We also found that adult platelets released higher concentrations of β2 M compared to neonatal platelets, both at rest and following activation, in agreement with the results of our recent proteomic study showing that adult platelets contain significantly more β2 M protein than neonatal platelets.^36^ Platelet-derived β2 M has been shown to induce a pro-inflammatory monocyte phenotype through binding to the monocyte TGF-β receptor (TGF-βr). Importantly, β2 M competes for binding at the TGF-βr with platelet released TGF-β, which conversely induces a pro-reparative monocyte phenotype, so that the balance between these two factors determines the ultimate platelet effect on monocytes.^17,18^ We found no differences in TGF-β release between neonatal and adult platelets, suggesting that -in balance- activated adult platelets might be more pro-inflammatory than neonatal platelets.

Since both P-selectin and β2 M have been shown to stimulate monocyte inflammatory responses, we hypothesized that the developmental state of the platelet would significantly influence the platelet-induced monocyte responses. In our co-culture system, neonatal monocytes stimulated with neonatal or adult platelets released similar concentrations of IL-8 and MCP-1, although there was a non-signficantlty higher cytokine release in response to adult platelets. An exploratory regression analysis showed a correlation between the monocyte IL-8 release and both platelet P-selectin expression and the concentration of β2 M in the platelet releasate, suggesting that differences in these platelet factors may be associated with differential levels of IL-8 production.

In addition to cytokine release, platelets induce a monocyte pro-migratory phenotype, characterized by increased surface expression of CCR2 and CCR5. Consistent with findings from a recent study of platelet transfusions given to thrombocytopenic newborn mice,^37^ we found that human neonatal monocytes exposed to adult platelets exhibited significantly higher surface expression of CCR2 and CCR5 compared to monocytes exposed to neonatal platelets. Increased CCR2 and CCR5 surface expression enables monocytes to migrate more readily in response to gradients in MCP-1 (CCL2) and RANTES (CCL5), the ligands for each receptor, respectively. Through this mechanism, platelet transfusions may amplify neonatal monocyte migration into inflamed tissues, such as the lungs in sick ventilated infants or the intestines in neonates with necrotizing enterocolitis, potentially mediating the reported associations between platelet transfusions and worsening lung disease and NEC.

In our co-culture system, blocking the interaction of P-selectin with its receptor (PSGL-1) completeley abrogated CCR5 receptor upregulation but not IL-8 and MCP-1 release by monocytes, suggesting that P-selectin is required to induce the monocyte pro-migratory phenotype but not pro-inflammatory cytokine production. These findings were consistent with published *in vivo* studies in newborn mice transfused with adult platelets, in which P-selectin blockade completely abrogated neonatal monocyte CCR2 and CCR5 upregulation but not the post-transfusion increase in the percentage of Ly6c^*hi*^ (pro-inflammatory) monocytes.^37^ Similarly, in a separate study, thrombocytopenic pups transfused with adult P-selectin^−/−^ vs. wildtype platelets exhibited similar increases in plasma pro-inflammatory cytokines 2–4-h post-transfusion, although only wildtype platelets increased circulating platelet-monocyte aggregates.^39^ Thus, the data suggest that the monocyte pro-migratory phenotype is regulated by platelet P-selectin exposure, which in turn is influenced by the platelet developmental stage. Monocyte cytokine release, however, is stimulated by platelets independently of surface P-selectin level or developmental stage. Similarly, a recent study found that platelets induce distinct functional programs in murine and human macrophages (specifically, ER stress and proinflammatory cytokine release) through separate pathways.^40^ Taken together, these data suggest that platelets are able to regulate multiple facets of the monocyte and macrophage responses through disctinct, non-overlapping, mechanisms. The role of developmental differences in platelet β2 M remains unclear, although our data suggest that β2 M alone is not sufficient to trigger cytokine production from neonatal monocytes.

Few studies have examined the immune and inflammatory effects of platelet transfusions in human neonates. One study comparing cytokine levels in dried whole blood spots of NICU patients before and after platelet transfusion found elevations in CXCL-5, CD40, and TGF-β post-transfusion.^41^ More recently, our group found significant elevations in the plasma concentrations of RANTES (CCL5), a potent chemokine implicated in multiple inflammatory disease processes, in NICU patients 2 to 4 hours after a platelet transfusion.^42^ The increase in RANTES is of particular interest in the context of the current study demonstrating that adult platelets upregulate the surface expression of CCR5, the RANTES receptor, on neonatal monocytes. Taken together, these studies suggest that an upregulation of the RANTES/CCR5 axis may be an important pathway mediating the pro-inflammatory, and potentially harmful, effects of platelet transfusions in neonates.

The convergence of results from *in vivo* murine studies and *in vitro* human studies is particularly important to confirm the biological relevance of the specific pathways through which the “developmental mismatch” associated with transfusing adult platelets into neonates dysregulates neonatal inflammatory responses in potentially harmful ways. A recent inter-species comparative study of the platelet proteome found that key platelet developmentally regulated pathways are conserved between mice and humans.^43^ At the individual protein level, this study identified a consistent increase in adult platelets across species of certain important inflammatory proteins, including β2 M, but not others, such as P-selectin. Upon activation, however, we found that neonatal platelets from both species displayed less surface P-selectin compared to adult platelets, albeit through different mechanisms. In humans, a neonatal degranulation defect results in decreased P-selectin translocation to the surface of activated neonatal platelets, while in mice it reflects lower P-selectin content in neonatal compared to adult platelets.^43^ The current study demonstrates that, despite inter-species differences in specific proteins, developmental differences in platelet function and P-selectin expression on activated platelets similarly impact monocyte responses across species.

This study is not without limitations. First, due to the impossibility of obtaining large enough volumes of neonatal blood to allow the isolation of peripheral blood monocytes, we utilized cord blood as an imperfect substitute for neonatal blood. While cord blood is neonatal in origin, the qualities of neonatal blood change over the course of the hours and days after delivery.^44,45^ Thus, it is possible that the monocyte inflammatory response of a 1-week-old infant could be different from that of its cord blood. In addition, all cord blood samples used in these studies were collected from full-term infants delivered via scheduled cesarean section. Thus, we were not able to investigate the response of monocytes from infants born premature due to the unpredictable timing of preterm birth. Since preterm infants are transfused platelets far more frequently than full term infants, this represents a limitation of our study. Finally, due to the small sample size, the secondary regression analyses presented in Table I should be considered exploratory and hypothesis-generating, rather than conclusive.

In conclusion, this study found key differences in the surface receptor expression of P-selectin and in the release of β2 M from neonatal versus adult platelets and discovered differential inflammatory responses from human neonatal cord blood monocytes after exposure to platelets of different developmental stages. These findings highlight the need to better understand platelet immune function over development to potentially modify, create, or choose platelet products that best match the developmental stage of our patients. In addition, our findings provide strong support to efforts aimed at avoiding unnecessary platelet transfusions, and raise questions about the safety of transfusing neonates with 15 mL/kg of platelets, a volume higher than that used in older children or adults.^46^

## Supplementary Material

Supp 1

Supp 2

Supplemental data for this article can be accessed online at https://doi.org/10.1080/09537104.2026.2726287

## Figures and Tables

**Figure 1.**
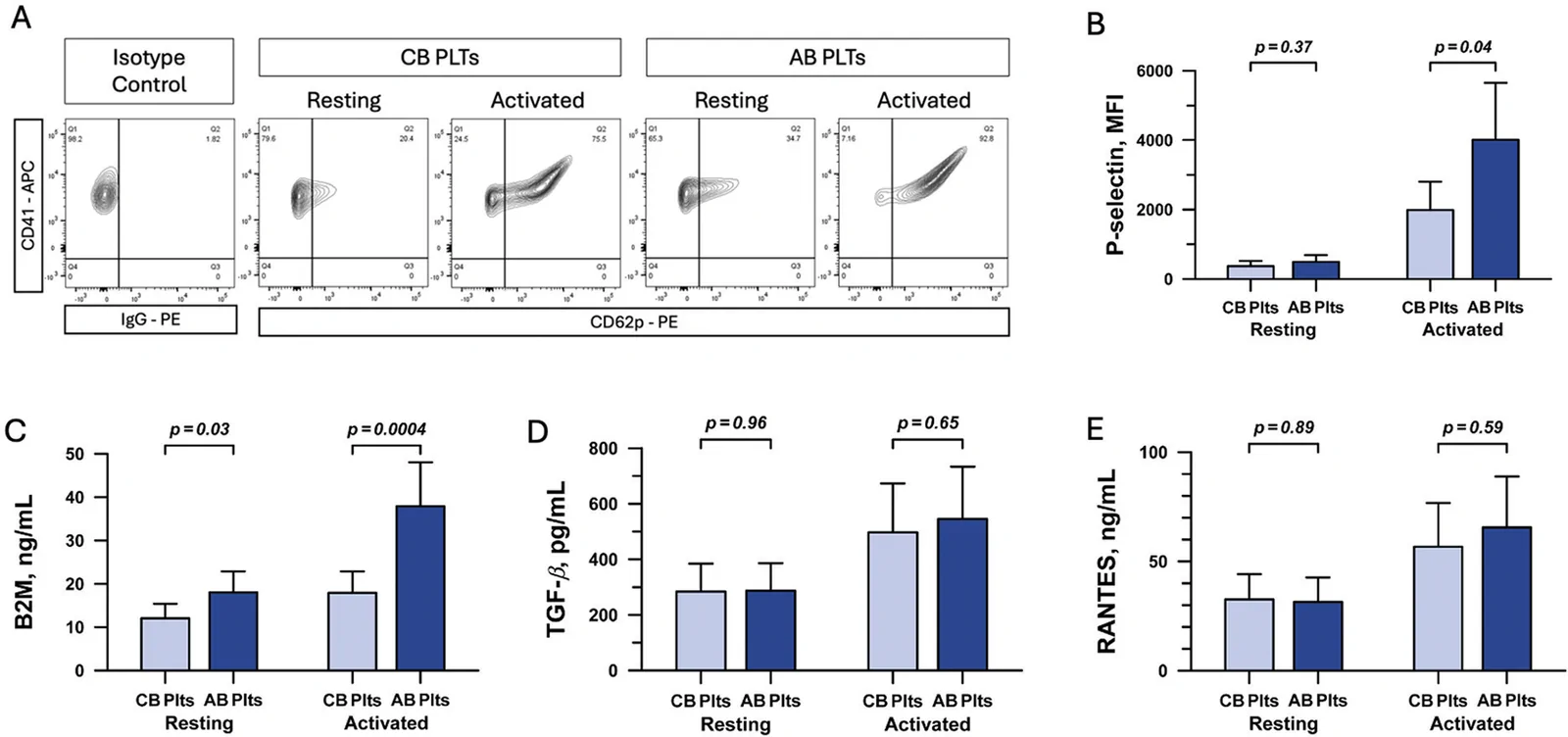
Representative flow plots of platelet CD62p surface expression in resting and activated cord blood (CB) and adult blood (AB) platelets compared to isotype control (A). Comparison of P-selectin expression (B), β2 M release (C), TGF-β release (D), and RANTES release (E) between resting and activated CB and AB platelets. Bars reflect the mean ± SE of 8 biologically independent samples per condition.

**Figure 2.**
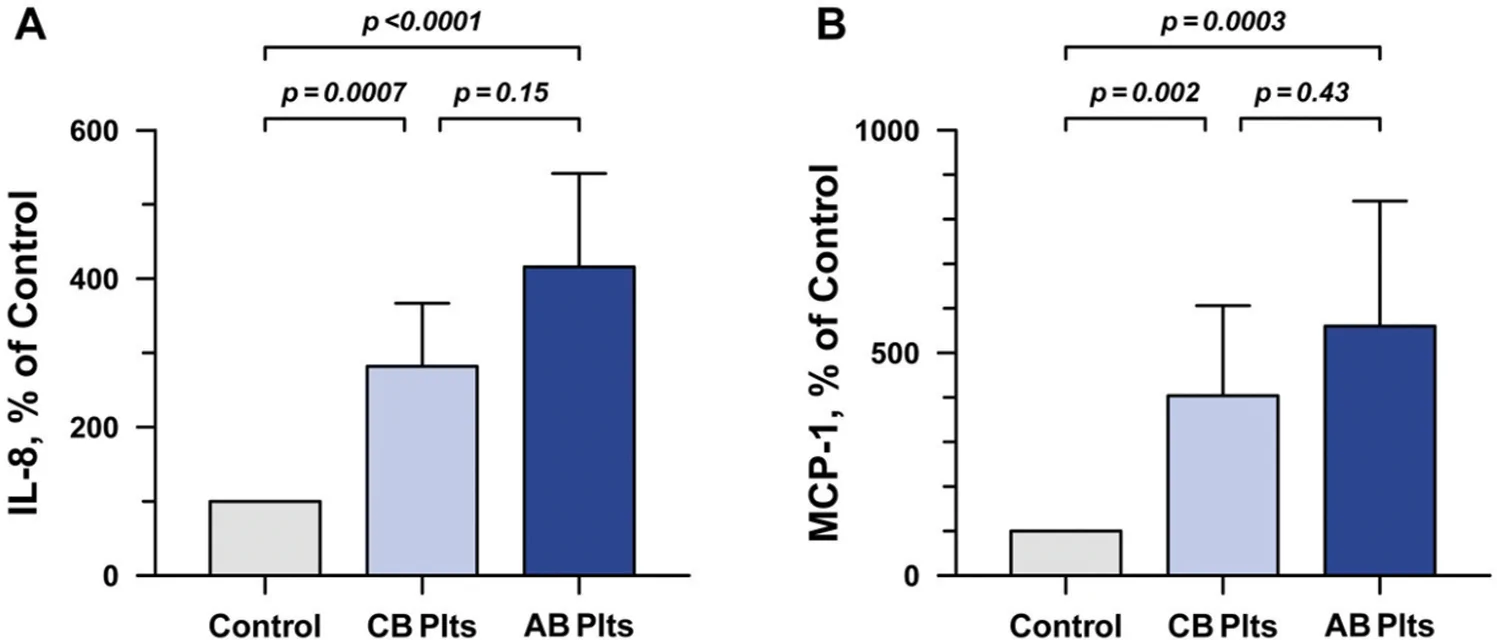
CB monocyte IL-8 (A) and MCP-1 (B) release after exposure to activated CB or AB platelets for 18 h. Results are expressed as % of control (monocytes alone). Bars reflect the mean ± SE of 12 cultures per condition.

**Figure 3.**
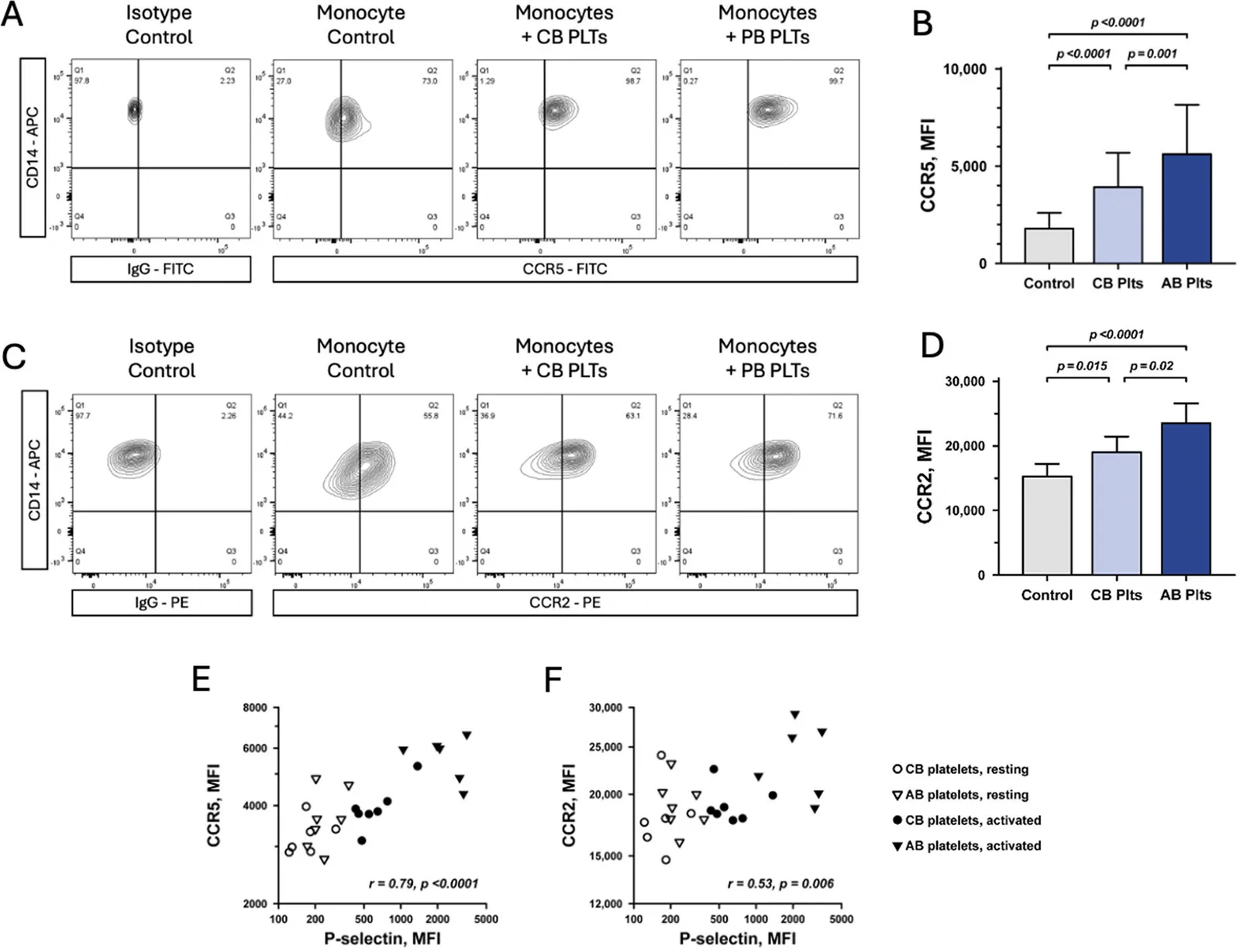
Representative flow plots of CB monocyte CCR5 surface expression without stimulation (control) or following exposure to activated CB or AB platelets for 4 h (A), CB monocyte CCR5 median fluorescent intensity (MFI) after co-culture (B), representative flow plots of CB monocyte CCR2 surface expression without stimulation (control) or following exposure to activated CB or AB platelet for 4 h (C), CB monocyte CCR2 MFI after co-culture (D). Correlation between monocyte CCR5 (E) and CCR2 (F) MFI and platelet P-selectin. *N* = 6 – 7 cultures per condition.

**Figure 4.**
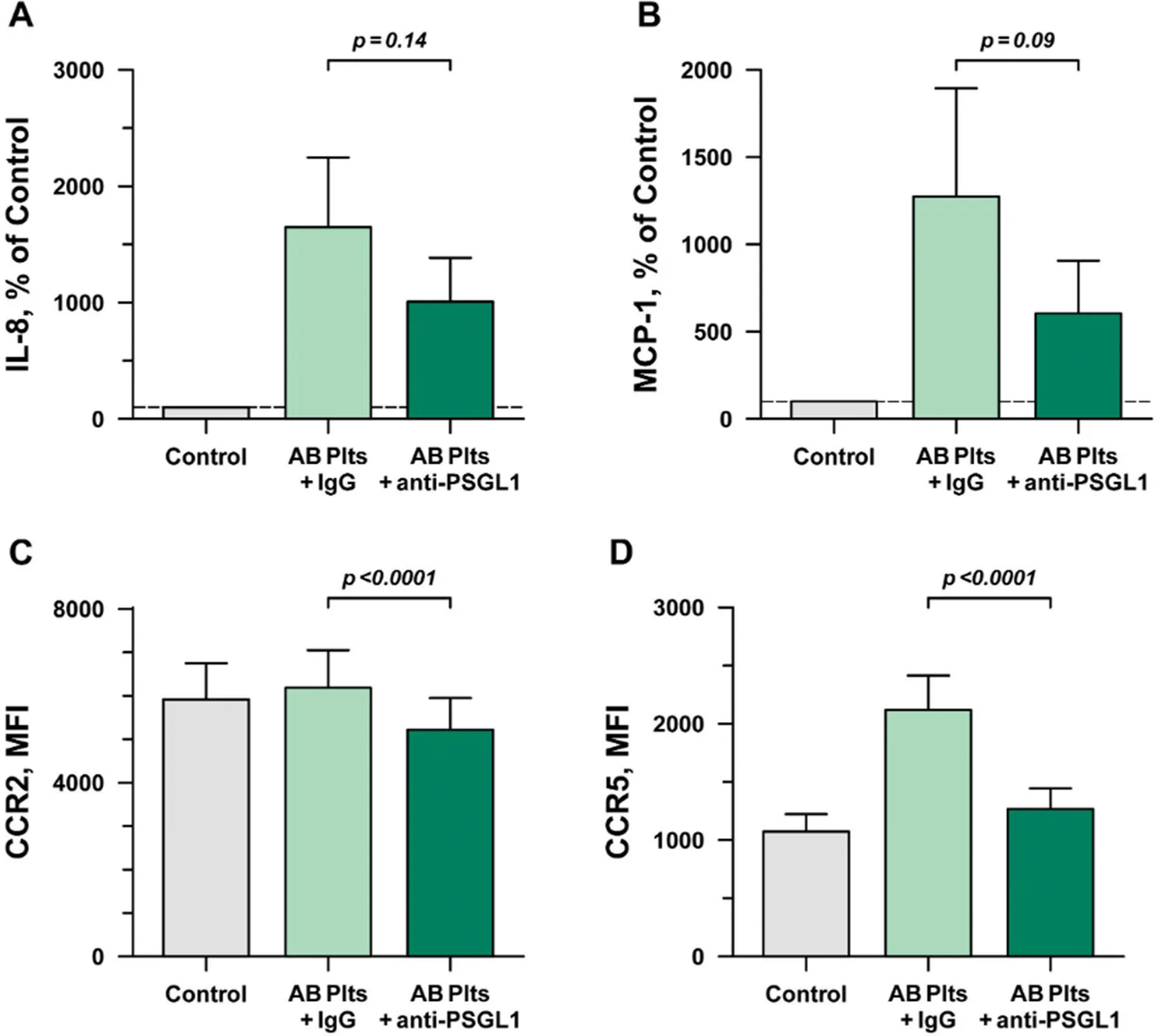
CB monocyte IL-8 (A) and MCP-1 (B) release and CCR2 (C) and CCR5 (D) surface expression after exposure to activated AB platelets with and without an anti-PSGL1 antibody. Cytokine values are expressed as % of control (monocytes alone) and surface receptors as measured MFI. Bars represent the mean ± SE of 8 cultures per condition.

**Table I.** Regression analysis.

CB Monocyte-derived cytokine (Y)	Platelet-derived factor (C)	Paired Samples	b ± SE*	p†

**IL-8**	**P-selectin**	**14**	**0.69 ± 0.12**	.**001**
	**β2 M**	**13**	**0.60 ± 0.22**	.**04**
	RANTES	13	1.23 ± 0.75	.16
	TGF-β	13	0.36 ± 0.45	.46
**MCP-1**	P-selectin	14	0.77 ± 0.33	.06
	β2 M	13	0.52 ± 0.53	.37
	RANTES	13	1.62 ± 1.31	.27
	TGF-β	14	−0.86 ± 0.73	.29

Note:

* Regression slope ± standard error. b = increase in log Y per unit log C resulting from addition of activated CB or PB platelets to CB monocytes, adjusted for log Y with thrombin alone. Additional analyses confirmed that effects of C were not mediated by age of blood donor. Multiple regression of log Y on log β2 M and log TGF-β (each controlled for the other) showed similar coefficients, standard errors, and significance levels to those obtain from analyzing each alone.

† Testing H_0_: b = 0.
