# Supplementary material for: Developmental differences in platelet P-selectin expression modulate neonatal monocyte inflammatory responses
Source: Platelets. Author manuscript; Available in PMC 2026 Nov 4. (PMC13732632; doi:10.1080/09537104.2026.2726287)
Supplement: Supplementary file 1 [file NIHMS2211765-supplement-Supp_1.pdf]

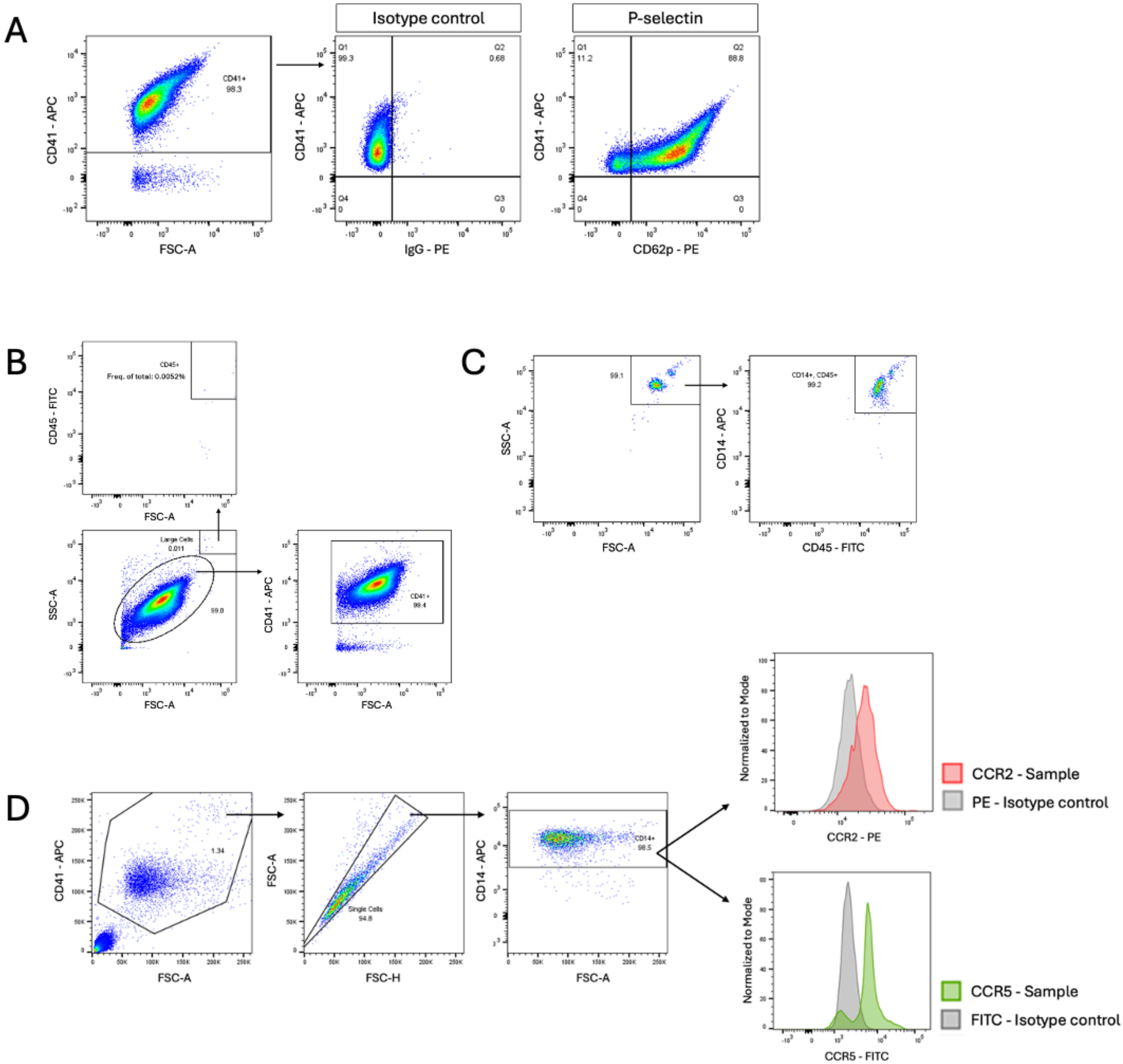
A
B
C
D
Isotype control
P-selectin
CD41+
98.3
Q1
99.3
Q2
0.68
Q3
0
Q4
0
Q1
11.2
Q2
88.8
CD41 - APC
FSC-A
IgG - PE
CD62p - PE
CD45+
Freq. of total: 0.0052%
CD45 - FITC
Large Cells
0.011
99.0
SSC-A
CD41+
99.4
99.1
CD14+, CD45+
99.2
CD14 - APC
CD45 - FITC
1.34
Single Cells
94.8
FSC-H
CD14+
98.5
CCR2 - PE
CCR5 - FITC
Normalized to Mode
CCR2 - Sample
PE - Isotype control
CCR5 - Sample
FITC - Isotype control
