# Supplementary material for: Developmental differences in platelet P-selectin expression modulate neonatal monocyte inflammatory responses
Source: Platelets. Author manuscript; Available in PMC 2026 Nov 4. (PMC13732632; doi:10.1080/09537104.2026.2726287)
Supplement: Supplementary file 2 [file NIHMS2211765-supplement-Supp_2.pdf]

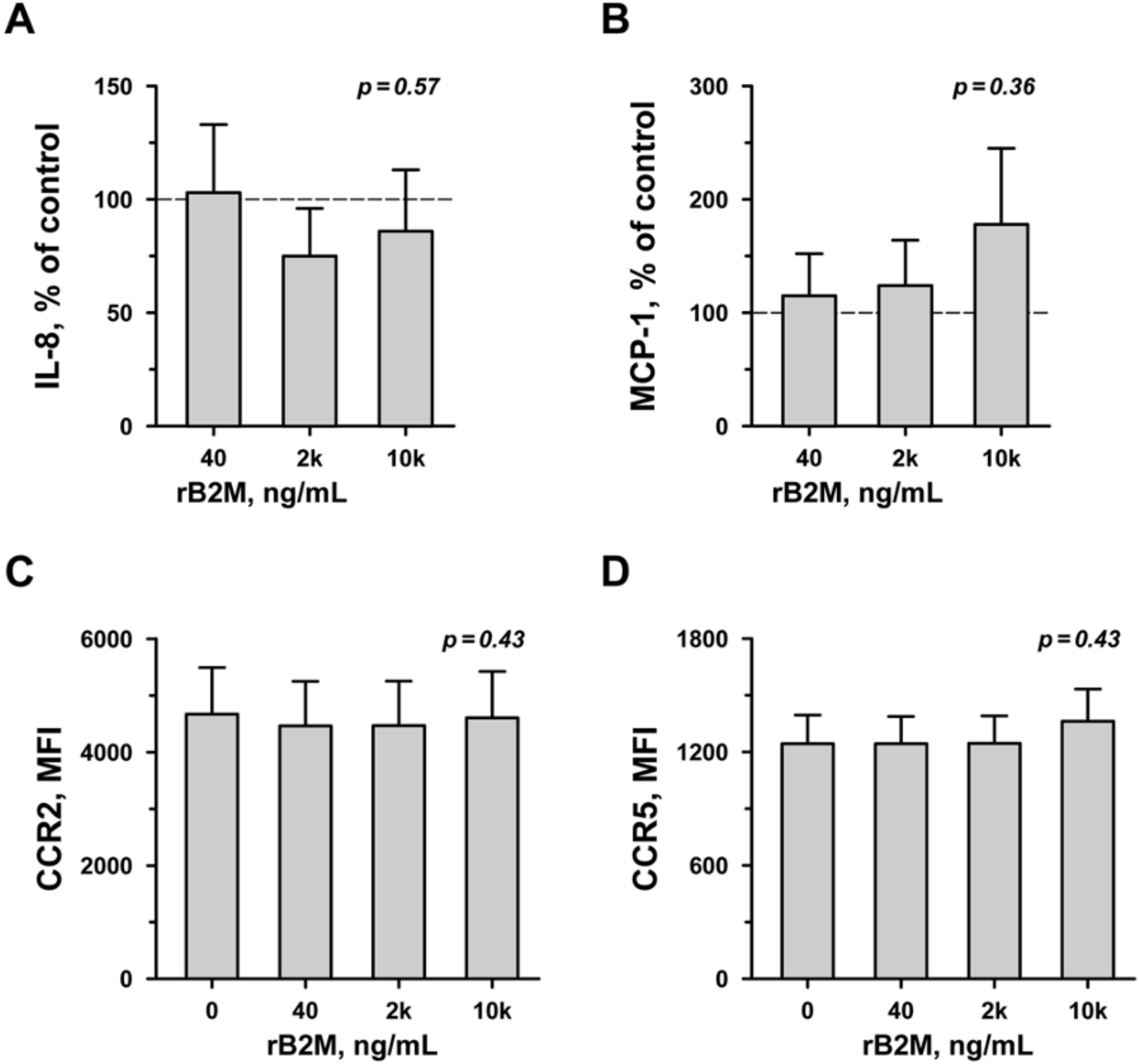
A
B
C
D
IL-8, % of control
MCP-1, % of control
CCR2, MFI
CCR5, MFI
p = 0.57
p = 0.36
p = 0.43
p = 0.43
150
100
50
0
300
200
100
0
6000
4000
2000
0
1800
1200
600
0
40
2k
10k
rB2M, ng/mL
